# N^6^-methyladenosine reader YTHDF1 regulates the proliferation and migration of airway smooth muscle cells through m^6^A/cyclin D1 in asthma

**DOI:** 10.7717/peerj.14951

**Published:** 2023-03-24

**Authors:** Juan Wang, Lei Wang, Xingfeng Tian, Lingping Luo

**Affiliations:** 1 Department of Nursing, Fenyang Colleage of Shanxi Medical University, Fenyang, China; 2 College of Nursing, Shanxi Medical University, Taiyuan, China; 3 Department of Student Affairs, Fenyang Colleage of Shanxi Medical University, Fenyang, China

**Keywords:** N6-methyladenosine, Asthma, Airway smooth muscle cells

## Abstract

Asthma is a chronic inflammatory respiratory disease, which is involved in multiple pathologic molecular mechanisms and presents a huge challenge to clinic nursing. Emerging evidence suggests that N^6^-methyladenosine (m^6^A) plays critical roles in respiratory system disease. Thus, present work tried to investigate the functions of m^6^A reader YTHDF 1 in asthma. The results indicated that YTHDF1 significantly upregulated in platelet-derived growth factor (PDGF) induced airway smooth muscle cells (ASMCs). Functionally, overexpression of YTHDF1 promoted the proliferation and migration of ASMCs, while YTHDF1 knockdown repressed the proliferation and migration. Mechanistically, there was a m^6^A modification site on cyclin D1 RNA (CCND1 genome) and YTHDF1 combined with cyclin D1 mRNA, thereby enhancing its mRNA stability via m6A-dependent manner. Collectively, these findings reveal a novel axis of YTHDF1/m6A/cyclin D1 in asthma’s airway remodeling, which may provide novel therapeutic strategy for asthma.

## Introduction

Asthma is well known as a common respiratory disorder, hyper-responsiveness accompanied by airway remodeling or chronic bronchial airway inflammatory (Ferry, De Castro & Bragg, 2020; Kutlu & Unal, 2019). In the pathophysiological process of asthma, the airway structures are involved, including airway cells, cell components and inflammatory substances (Lee, Lee & Hong, 2020; MacDonald & Barrett, 2019). The pathogenesis of asthma is predominantly attributed by immune cells’ excessive immune response and substantial amounts of inflammatory cytokines production, *e.g.*, interleukin 4/5/13. These cytokines mediate mucus and immunoglobulin E overproduction, airway hyperresponsiveness and eosinophilic infiltration (Sánchez et al., 2019). Thus, the most effective way to deal with asthma is to discover its precise pathogenesis.

N^6^-methyladenosine (m^6^A) methylation is a post-transcriptional RNA modification epigenetically occurred on mRNAs, which regulates gene expression and affects the RNA fate (Liu et al., 2021; Sweaad et al., 2021; Tuncel & Kalkan, 2019). Presently, m^6^A methylation gains more and more attention about its function and mechanism. In the asthma pathophysiological process, there is only primary probation and initiatory findings (Wang et al., 2021; Wu et al., 2021). For example, Teng et al. (2021) found that dysregulated or hypermethylated m^6^A peaks in 329 mRNAs and 150 hypomethylated m^6^A peaks in 143 mRNAs in asthmatic mice. In addition, Dai et al. (2021) found that 5 candidate m^6^A regulators (FMR1, KIAA1429, WTAP, YTHDF2, ZC3HAV1) are in close contact with the risk of childhood asthma. Therefore, these literatures inspire us that m^6^A may participate in the asthma pathology.

In clinical nursing, the particularity of asthma brings great challenge to nursing work. Some environmental factors can trigger or stimulate asthma, such as pollen, excessively cold climate, strenuous exercise or pets (Sonney et al., 2022). Asthma can cause recurrent episodes of wheezing, shortness of breath, chest tightness, and/or coughing (Yamada et al., 2022). In addition, these emergencies often occur at night/morning. Therefore, this special situation requires us to pay close attention to the patient’s changes during the course of nursing.

Here, our research found that, in the cellular asthma model induced by PDGF-BB, the m^6^A modification significantly varied and the m^6^A regulator key-enzymes also altered. To investigate the potential roel of m^6^A in asthma, we focused on a critical m^6^A reader YTHDF1. Results indicated that YTHDF up-regulated in the PDGF-BB induced ASM cells. Functionally, YTHDF1 posituvely promoted the proliferation and migration of ASM cells. Interestingly, an important element cyclin D1 (CCND1) acted as the downstream target of YTHDF1 *via* m^6^A-depedent manner. Mechanistically, YTHDF1 significantly combined with cyclin D1 mRNA, thereby enhancing its mRNA stability through m^6^A-depedent pattern, which may provide novel therapeutic strategy for asthma.

## Materials and methods

### Asthmatic cellular model

As previously described (Ba et al., 2018), the primary cultured human ASM cells were obtained from 2nd–4th generation mainstem bronchi of patients undergoing lung resection surgery in accordance with procedures. Written informed consent was obtained from every human participant. The assay had been approved by the Ethics Committee of Shanxi Medical University (No. SXMU201905047). In brief, the mainstem bronchi segments were cut into pieces and ASMCs were isolated from it. After digestion, ASMCs were placed in DMEM medium containing 10% fetal bovine serum (Gibco, NY, USA) in humidified incubator at 5% CO_2_ 37 °C with. Cells from passages 4–7 were used for following assays, ASMCs were treated with PDGF-BB (25 ng/ml) to mimic the asthma.

### Transfection

For the transfection of YTHDF1, ASMCs were transfected with sequences following the manufacturer’s instructions. For silencing of YTHDF1, the shRNA sequences of YTHDF1 were synthesized by OBiOc (Shanghai, China) and the vectors containing shRNAs were inserted into PLKO.1. The transfection of plasmids was performed using the Lipofectamine 3000 kit (Invitrogen) according to the manufacturer’s instructions. For the overexpression of YTHDF1, the full-length cDNA sequences of human YTHDF1 (gene ID: 54915) were cloned into a pLentiEF1a-EGFP-Puro-CMV-MCS-3Flag lentivirus vector. The transfection efficiency was evaluated with qRT-PCR or western blot.

### Reverse transcription quantitative polymerase chain reaction (RT- qPCR)

Total RNA was extracted according to the instruction of HiScript II 1st Strand cDNA Synthesis Kit (Vazyme, Nanjing, China). Then, cDNA was synthesized using an PrimeScript RT reagent kit (Takara, Dalian, China). Real-time PCR was performed on the 7900 Real-time PCR System using Taqman RNA assay kit (Thermo Fisher Scientific, Rockford, IL, USA). Glyceraldehyde-3-phosphate dehydrogenase (GAPDH) acted as a control. The primer sequences were listed in Table S1. The results of transcript levels were analyzed by the 2^−ΔΔCt^ method.

### Western blot analysis

Total protein in ASMC cells was extracted using radio immunoprecipitation assay (RIPA) lysis buffer with phenylmethanesulfonyl fluoride (PMSF) (Solarbio, Beijing, No. R0010). After incubation on ice and then concentration, the protein concentration was measured by bicinchoninic acid (BCA) kit and adjusted by deionized water. Protein samples were separated by sodium dodecyl sulfate-polyacrylamide gel electrophoresis (10% SDS-PAGE) and transferred to polyvinylidene fluoride membranes (PVDF) (Millipore, Billerica, MA, USA). The transferred PVDF membranes (No. ISEQ00010; Millipore, Billerica, MA, USA) was added with Tris-buffered saline tween (TBST) containing 5% dried dskimmed milk. Then, PVDF membranes were incubated with the primary antibodies (anti-Cyclin D1, ab16663; Abcam; anti-YTHDF1, 1:1000, ab220162; Abcam). Beta-actin (1:1,000; Abcam) was used as an internal control. After washing with phosphate buffer five times containing Tween-20 (PBST), PVDF membranes were incubated for 1 h at room temperature. Finally, pierce ECL western blotting substrate was employed to develop the protein bands and quantification was conducted by Image Lab software (Bio-Rad, Hercules, CA, USA).

### Proliferation assays and cycle analysis

The proliferation of ASMCs was detected using CCK-8 assays using Cell counting kit-8 (8 µl of CCK-8; Dojindo, Kumamoto, Japan) with 100 µl serum free medium and incubated for 90 min. In brief, the transfected ASMCs (5 ×10^3^ cells/well) was inoculated at into 96-well plates at 24, 48, and 72 h of culture at 37 °C overnight. Eventually, the absorbance at 450 nm was detected by a microplate reader (Thermo Fisher Scientific, Waltham, MA, USA). For the cycle analysis, Coulter EPICS XL flow cytometer (Beckman Coulter, Inc., Fullerton, CA, USA) was performed by flow cytometry on flow cytometry with Modifit software (BD Biosciences).

### Transwell migration assays and wound healing assay

The migration of ASMCs was detected by transwell assays. In brief, the transfected ASMCs (1 ×10^5^ cells/well) were suspended in serum-free medium upper transwell chamber (pore size: 8 µm; Corning, Inc., Corning, NY, USA). In the bottom chamber, medium was supplemented with 600 µl of 10% FBS. After being incubated for 24 h at 37 °C, the cells in the top compartment were wiped off by cotton swabs and the migrated cells were fixed for 20 min with 4% paraformaldehyde and stained for 10 min with 0.1% crystal violet staining solution (Sigma-Aldrich, Louis, MO, USA). Lastly, images were taken under the optical microscope (Olympus, Tokyo, Japan). For the wound healing assay, the monolayer was manually scraped by sterile pipette tip. After being 24 h incubation at 37 °C, the Images of wound closure were evaluated by inverted microscope (Olympus, Tokyo, Japan). The migration rate was calculated by the formula: migration rate = migration distance/original distance.

### RNA immunoprecipitation (RIP)-PCR

The interaction within RNA binding proteins and mRNA was identified using RIP-PCR. In brief, the RIP experiment was carried out by EZ-Magna RIP Kit (Millipore) according to the manufacturer’s protocol. ASMCs were lysed in complete RIP lysis buffer, and the cell extract was incubated with protein A/G agarose beads conjugated by anti-YTHDF1 (ab220162, 1:30; Abcam) or control IgG (ab172730; Abcam) for 2 h at 4 °C. After being washed, beads were incubated with Proteinase K to remove protein in complex. Lastly, the purified RNAs were subjected to qRT-PCR analysis.

### RNA stability assay

To detect the cyclin D1 mRNA stability, the ASMCs were treated with actinomycin (Act D, 2 µg/mL) treatment for 0, 3 and 6 h. The relative remaining RNA level was detected by qRT-PCR and the half-life of cyclin D1 mRNA was examined by transcript levels at indicated time points relative to those before Act D treatment.

### Luciferase reporter assay

The wild-type or mutant Cyclin D1 3′-UTR was synthesized and inserted into pmirGLO reporter vector (Promega, Madison, WI, USA), and then co-transfected with WT- Cyclin D1 or Mut- Cyclin D1 pcDNA 3.0 expressing plasmid into cells using Lipofectamine 3000. Cells were transfected with pGL3-Luc (1 µg) as a control for transfection efficiency (Promega), according to the manufacturer’s instructions. The luciferase activity was tested using a luciferase reporter commercial kit (Promega, Madison, WI, USA).

### Statistical analysis

Experiments were repeated three times and data was presented as means ± standard deviation (SD) in this study. Variables between was compared by student’s *t*-test and

Statistical analyses were performed using the SPSS 20.0 software (SPSS, Inc., Chicago, IL, USA) and GraphPad Prism 8.0 software (GraphPad, San Diego, CA, USA). A two-sided *p*-value of less than 0.05 was considered statistically significant.

## Results

### YTHDF1 was up-regulated in the PDGF-induced ASMCs

**Figure 1:**
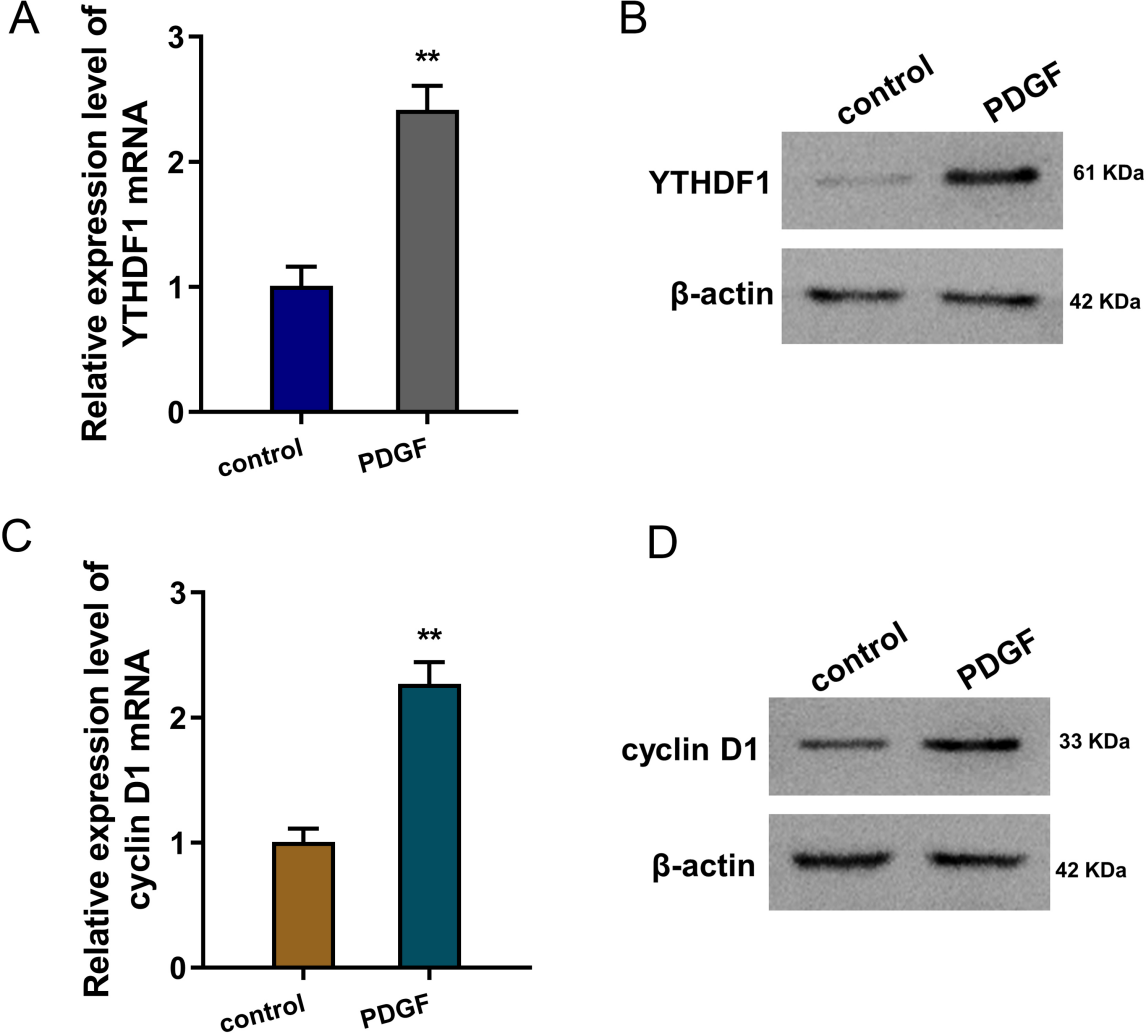
YTHDF1 was up-regulated in the PDGF-induced ASMCs. (A) RT-qPCR analysis was performed to detect the YTHDF1 mRNA level in the PDGF-induced ASMCs and control group. (B) Western blot analysis was carried out to measure the YTHDF1 protein level in the PDGF-induced ASMCs. (C) The cyclin D1 mRNA level was detected using RT-qPCR analysis in the PDGF-induced ASMCs. (D) The cyclin D1 protein level was detected using western blot analysis in PDGF-induced ASMCs. **p < 0.01.

To mimic the cellular asthma model, PDGF-induced ASMCs and blank control cells were constructed. Firstly, we found that YTHDF1 mRNA was up-regulated in the PDGF-induced ASMCs (Fig. 1A). Besides, the YTHDF1 protein level also up-regulated in the PDGF-induced ASMCs (Fig. 1B). Moreover, we found cyclin D1 (CCND1), an essential cell cycle control gene closely correlated to the development of asthma (Thun et al., 2013), was up-regulated in the PDGF-induced ASMCs (Fig. 1C). Besides, the cyclin D1 protein level also up-regulated in the PDGF-induced ASMCs (Fig. 1D). Overall, these findings revealed that YTHDF1 was up-regulated in the PDGF-induced ASMCs.

### YTHDF1 positively regulated the proliferation and migration of ASMCs

**Figure 2:**
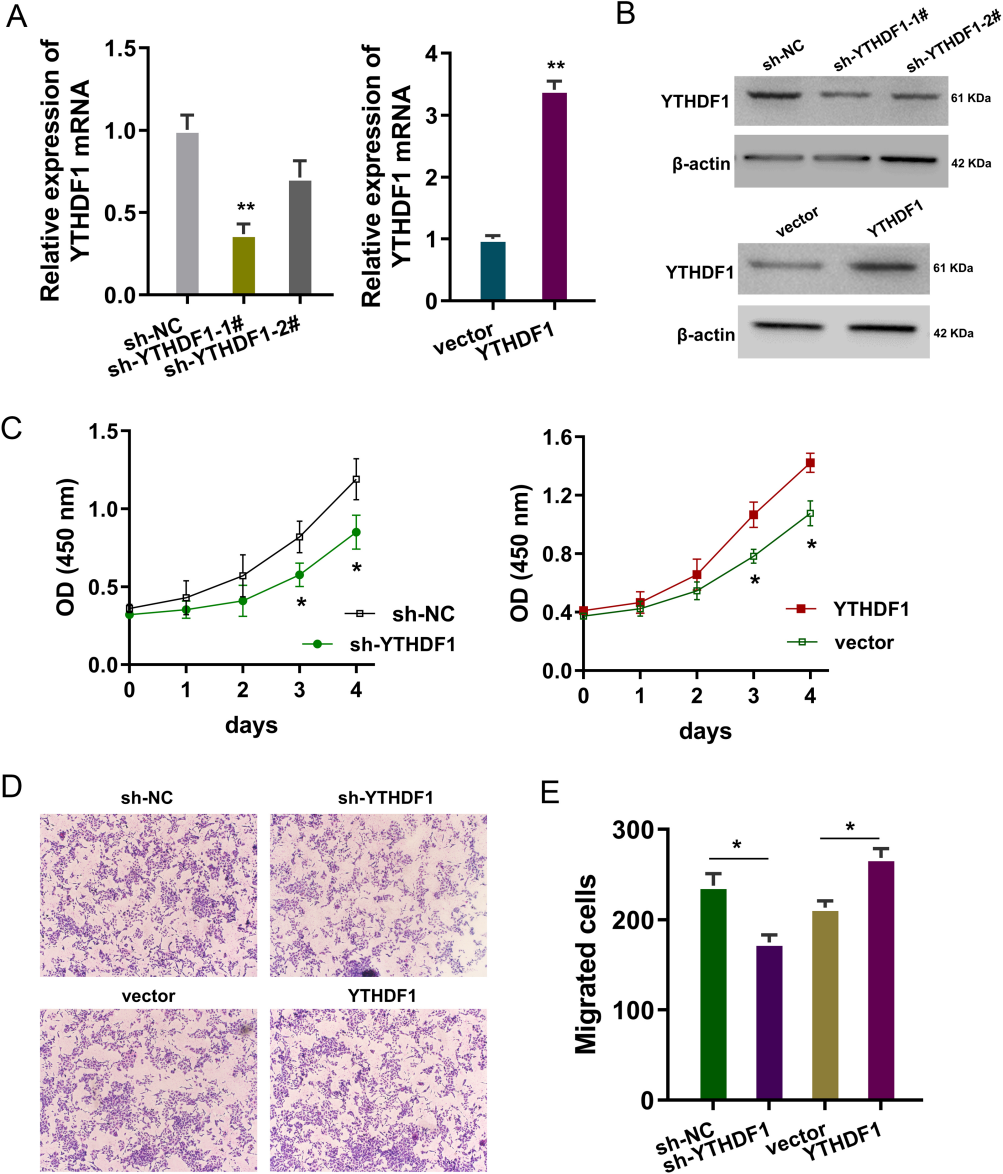
YTHDF1 positively regulated the proliferation and migration of ASMCs. (A) The transfection efficiency for YTHDF1 was detected using RT-PCR and (B) western blot. Results reflected the YTHDF1 mRNA and YTHDF1 protein after YTHDF1 silencing (sh-YTHDF1-1#/2#, sh-NC) and YTHDF1 enforced overexpression (YTHDF1, vector). (C) Proliferative ability of ASMCs was detected by CCK-8 assay after YTHDF1 silencing (sh-YTHDF1-1#/2#, sh-NC) and YTHDF1 enforced overexpression (YTHDF1, vector). (D, E) The migrative ability of ASMCs was determined by transwell assay. *p < 0.05; **p < 0.01.

The bio-functional roles of YTHDF1 were explored in the PDGF-induced ASMCs with YTHDF1 knockdown and overexpression. The transfection efficiency was detected using RT-PCR (Fig. 2A) and western blot (Fig. 2B). Proliferation analysis by CCK-8 assay unveiled that YTHDF1 silencing repressed the proliferative ability of ASMCs, while the YTHDF1 enforced overexpression up-regulated the proliferative ability (Fig. 2C). Furthermore, the migrative ability of ASMCs was determined by transwell assay, and results illustrated that YTHDF1 silencing repressed the migrative ability of ASMCs, while the YTHDF1 enforced overexpression up-regulated the migrative ability (Figs. 2D, 2E). Overall, these findings revealed that YTHDF1 positively regulated the proliferation and migration of ASMCs.

### YTHDF1 positively facilitated the cell cycle progression and migration

**Figure 3:**
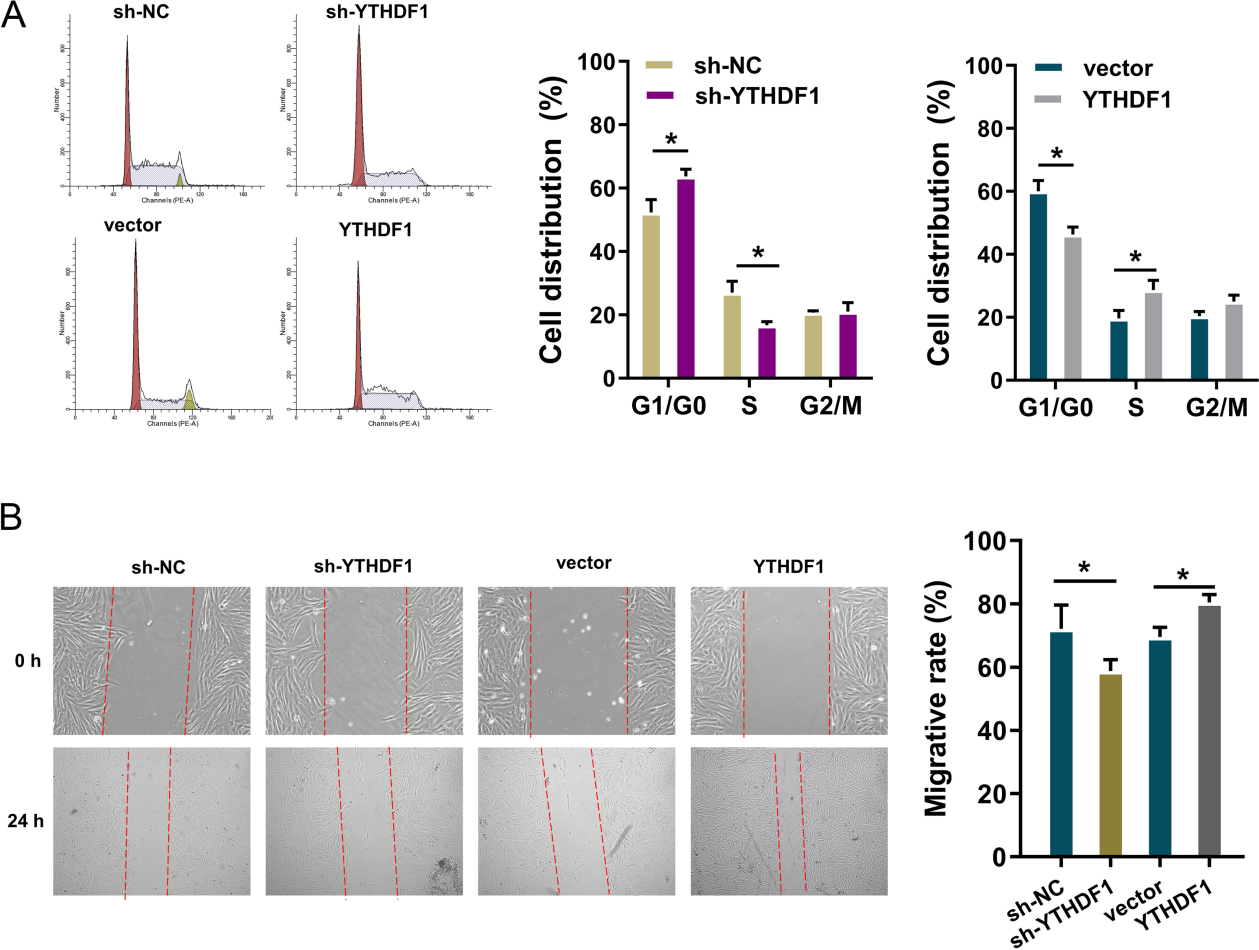
YTHDF1 positively facilitated the cell cycle progression and migration. (A) Flow cytometry cell cycle analysis was performed in PDGF-induced ASMCs with YTHDF1 knockdown (sh-YTHDF1) or control (sh-NC). (B) Wound healing assay was performed to determine the the migrative ability of ASMCs with YTHDF1 knockdown (sh-YTHDF1) or control (sh-NC). *p < 0.05; **p < 0.01.

To detect the role of YTHDF1 on PDGF-induced ASMCs, flow cytometry cell cycle analysis was performed. The results showed that YTHDF1 knockdown induced the cycle arrest at G1/S phase, while YTHDF1 overexpression promoted the cycle progression of ASMCs (Fig. 3A). Furthermore, the migrative ability of ASMCs was determined by wound healing assay, and results illustrated that YTHDF1 knockdown repressed the migrative ability of ASMCs, while the YTHDF1 overexpression up-regulated the migrative ability (Fig. 3B). Therefore, these data showed that YTHDF1 positively facilitated the cell cycle progression and migration.

### Cyclin D1 acted as the target of YTHDF1

**Figure 4:**
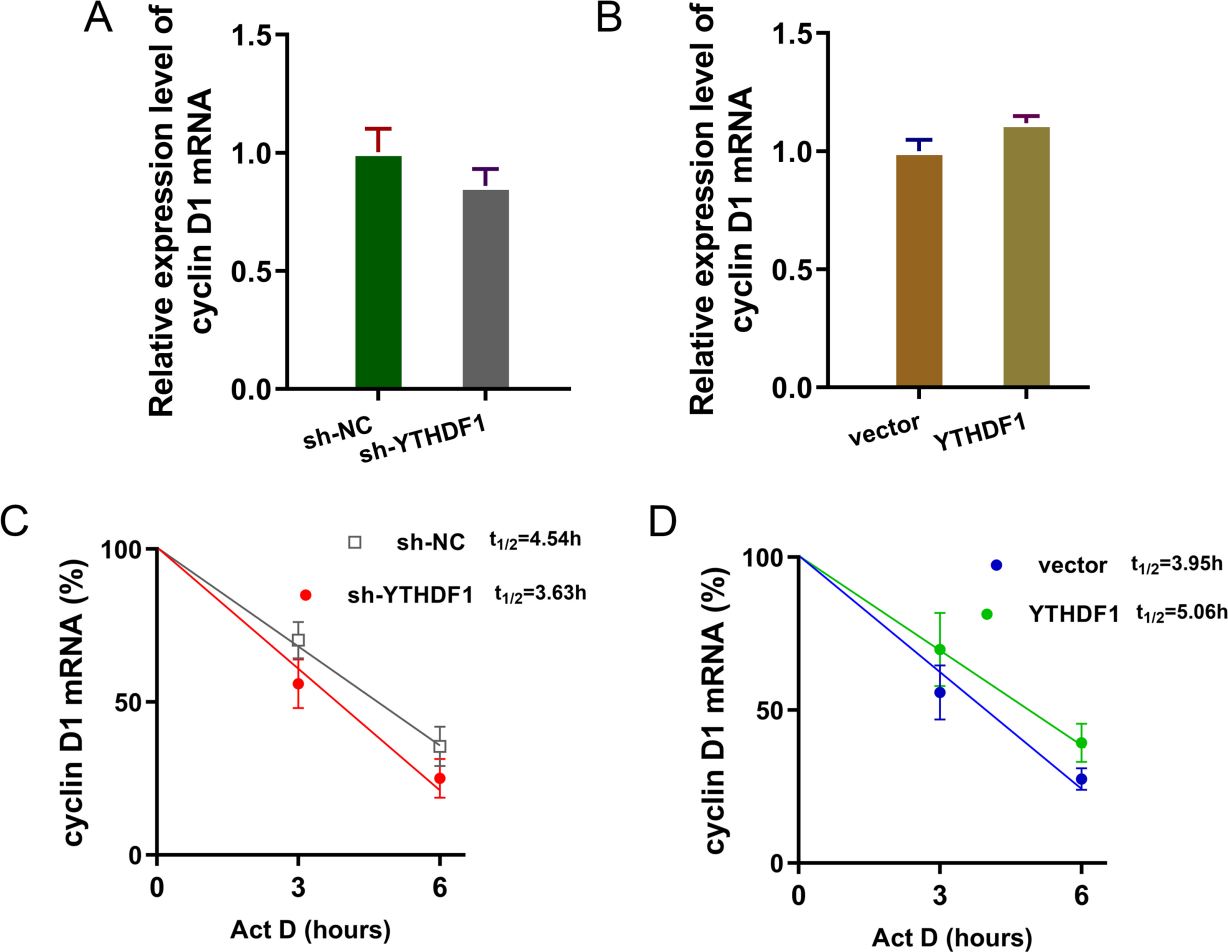
Cyclin D1 acted as the target of YTHDF1. (A) The predictive tool (SRAMP, http://www.cuilab.cn/sramp) was utilized to analyze the m^6^A site in these potential downstream targets of YTHDF1 in asthma. (B) The exact site of m^6^A modification (ATGGAC) on the Cyclin D1 gene. (C) The m^6^A motif on the Cyclin D1 mRNA was predicted (https://rna.sysu.edu.cn/rmbase/). (D) RIP-PCR was performed to determine the molecular interaction within Cyclin D1 and YTHDF1 in PDGF-induced ASMCs (*p* = 0.004). (E) RIP-PCR was performed in ASMCs transfected with YTHDF1 silencing (sh-YTHDF1-1#/2#, sh-NC) and YTHDF1 enforced overexpression (YTHDF1, vector) (*p* = 0.038). *p < 0.05; **p < 0.01.

To discovery the potential downstream target of YTHDF1 in asthma, we utilized the predictive tool (SRAMP, http://www.cuilab.cn/sramp) to analyze the m^6^A site in these targets (Fig. 4A). Moreover, we found that the exact site of m^6^A modification (ATGGAC) on the Cyclin D1 gene (Fig. 4B). The m^6^A motif on the Cyclin D1 mRNA was predicted (https://rna.sysu.edu.cn/rmbase/) (Fig. 4C). The molecular interaction within Cyclin D1 and YTHDF1 was determined by RIP-PCR, and results indicated that YTHDF1 closely combined with Cyclin D1 (Fig. 4D). Moreover, further RIP-PCR analysis found that YTHDF1 silencing repressed the combination within Cyclin D1 and YTHDF1, and YTHDF1 overexpression enhanced the combination (Fig. 4E). Taken together, these findings revealed that Cyclin D1 acted as the target of YTHDF1.

### YTHDF1 enhanced the RNA stability of cyclin D1 mRNA *via* m6A-dependent manner

**Figure 5:**
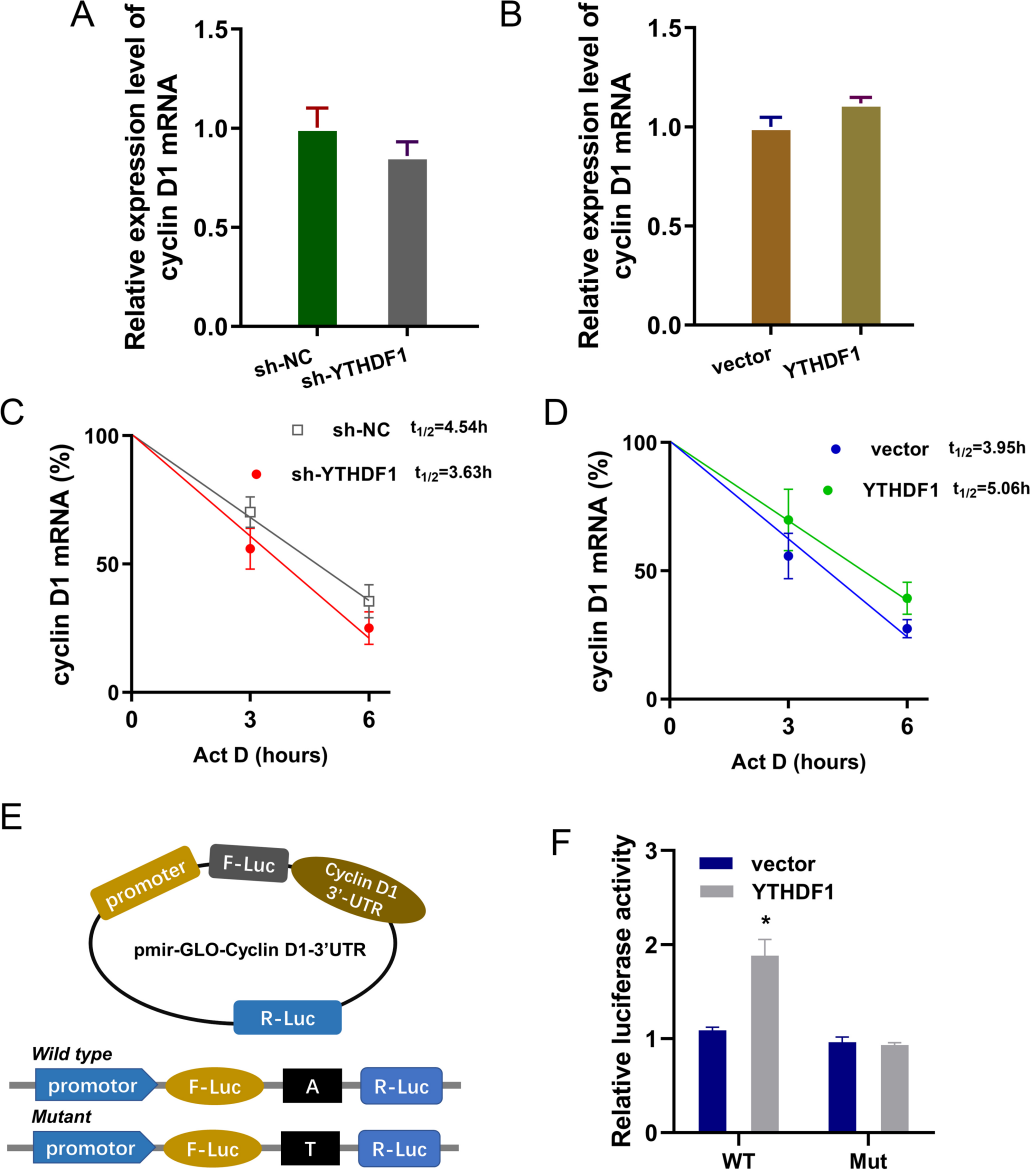
YTHDF1 enhanced the RNA stability of cyclin D1 mRNA. (A, B) RT-PCR analysis was performed to detect the cyclin D1 mRNA level in ASMCs with YTHDF1 silencing or overexpression. (C, D) RNA stability analysis was performed to revealed the half life time (t_1/2_) of cyclin D1 mRNA in ASMCs upon Act D treatment. (E, F) cyclin D1 mRNA 3′ UTR containing m^6^A modification site was cloned into luciferase reporter vectors, including mutation (Mut) of m6A consensus sequence and mutant by replacing adenosine with cytosine. The luciferase activity within cyclin D1 wild type sequence and YTHDF1 overexpression or control was detected. Relative luciferase activity was computed by the ratio of Firefly and Renilla luciferase values.

**Figure 6:**
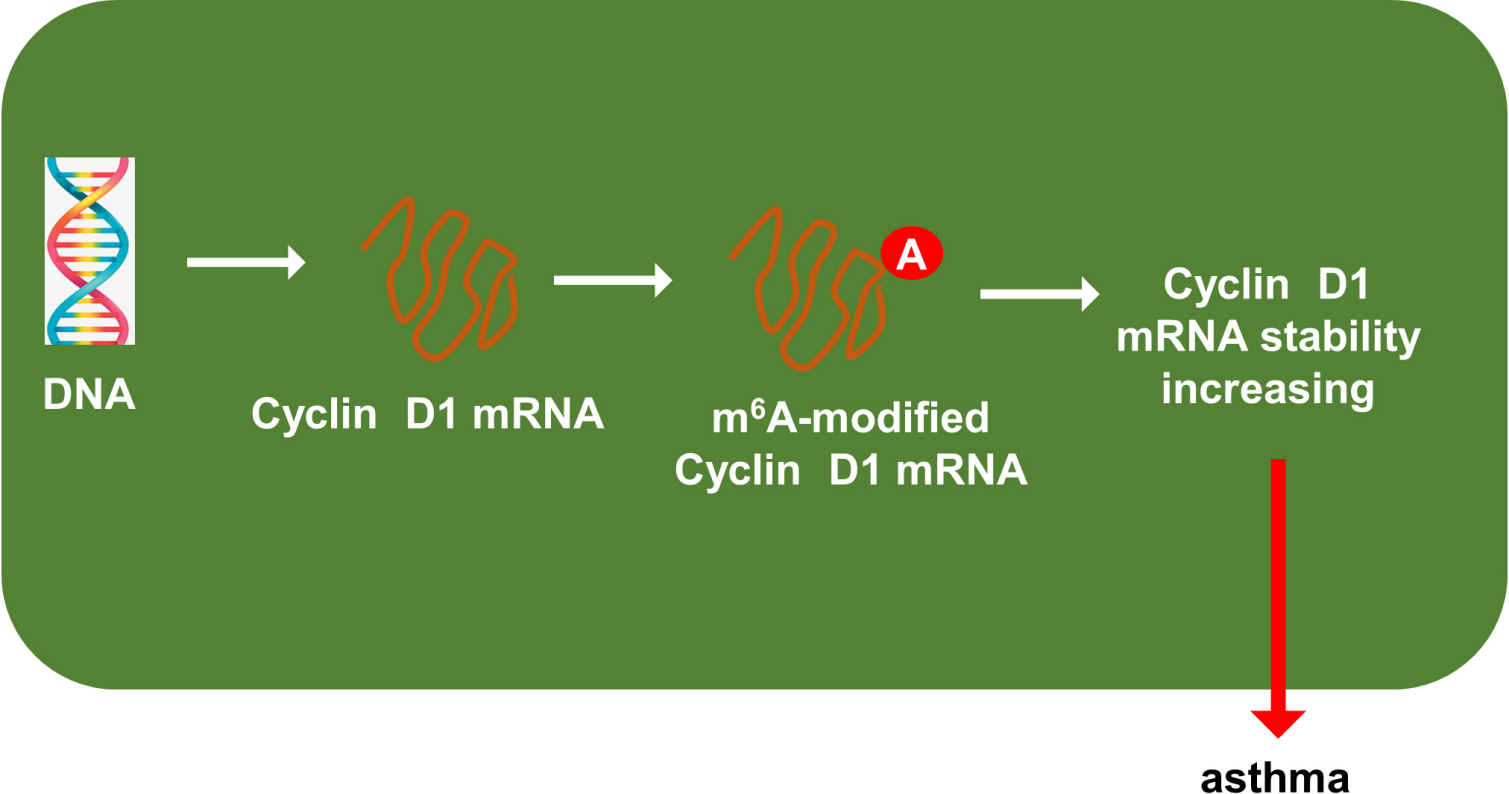
YTHDF1 enhanced the RNA stability of cyclin D1 mRNA to positively regulate asthma.

Previous researches had revealed that YTHDF1 could recognize the m^6^A site on mRNA and then enhance its stability (Chen et al., 2021; Xu et al., 2021). In our study, we found that YTHDF1 might also target cyclin D1 mRNA to enhance its stability. Firstly, we detected the cyclin D1 mRNA level in ASMCs with YTHDF1 silencing or overexpression, and results showed that YTHDF1 silencing or overexpression didn’t significantly regulate the cyclin D1 mRNA (Figs. 5A, 5B). RNA stability analysis revealed that YTHDF1 silencing reduced the half life time (t_1/2_) of cyclin D1 mRNA upon Act D treatment, while YTHDF1 overexpression up-regulated the half life time (t_1/2_) of cyclin D1 mRNA (Figs. 5C, 5D). Luciferase reporter assay using wild-type (WT) cyclin D1 3′UTR or mutant (Mut) was performed and results indicated that YTHDF1 accelerated the luciferase activity within cyclin D1 wild type (Figs. 5E, 5F). In conclusion, we found that YTHDF1 enhanced the RNA stability of cyclin D1 mRNA *via* m^6^A-dependent manner (Fig. 6).

## Discussion

Currently, the pathophysiology of asthma is complex, including airway remodeling and inflammatory cells invasion and so on. Airway remodeling refers to a series of structural changes in airway structure in patients with asthma, including epithelial injury, increased basement membrane thickness, airway smooth muscle thickening, goblet cell metaplasia, and airway vascular and lymphatic proliferation (Xu et al., 2022). This study unveiled a novel finding that m^6^A reader YTHDF1 play acritical role in asthma airway remodeling, involving ASMCs proliferation and migration abilities.

N^6^-methyladenosine (m^6^A) is the most abundant modification in mRNA, which is regulated by m^6^A methyltransferases, demethylases and readers. In the respiratory diseases, there are more and more literature reveal the important functions of m^6^A *via* variety of evidence (Xu et al., 2022). For instance, m^6^A writer METTL3 is up-regulated in PM2.5 exposured mice lung injury and METTL3 up-regulated the m^6^A modification of Interleukin 24 (IL24) through *via* METTL3/YTHDF1-coupled epitranscriptomal regulation (He et al., 2022). In lung ischemia/reperfusion injury, the m^6^A reader YTHDF3 or IGF2BP2 knockdown attenuates the hypoxia/reoxygenation-mediated inhibitory effects in BEAS-2B cells, as well as the hypoxia/reoxygenation-induced cell apoptosis (Xiao et al., 2022). Thus, these findings show the important function of m^6^A modification in respiratory diseases.

Here, present research indicated that a novel m^6^A reader YTHDF1 also significantly up-regulated in the asthma cellular model. In the PDGF-induced ASMCs, we found that m^6^A reader YTHDF1 up-regulated and the functional assays suggested that YTHDF1 overexpression promoted the proliferation and migration of ASMCs. Thus, our assays’ data revealed the potential function of m^6^A reader YTHDF1 in asthma.

Moreover, we found that a important element cyclin D1 (CCND1) up-regulated in the asthma cellular model (PDGF-induced ASMCs). Mechanistically, there were remarkable m^6^A modified site on cyclin D1 mRNA. Then, RIP-PCR assays was performed and results indicated that YTHDF1 significantly combine with cyclin D1 mRNA, thereby enhancing its mRNA stability through m^6^A-depedent manner. Collectively, these findings reveal a YTHDF1/cyclin D1 axis in asthma.

As regarding the role of cyclin D1 (CCND1), an essential cell cycle control gene, convictive literature has revealed that CCND1 is closely correlated to the development of asthma (Thun et al., 2013). Besides, Li et al. (2021) reported that cell cycle regulation may play a role in asthma initiation and development, and CCND1 rs9344 genotype serves as an early detection marker for asthma. Overall, we could conclude that cyclin D1 (CCND1) significantly participate in the asthma.

Asthma is a chronic inflammation; however, this inflammation is not primarily caused by bacterial infections. Asthma attacks are mostly related to exposure to allergens, cold air, physical and chemical stimulation, emotional changes, respiratory tract infection and exercise (Zhou et al., 2021). Thus, the complex pathogenesis put forward higher request to clinical nursing. For the clinical nursing of asthma, there are many situations that require protection, such as the allergies. To eliminate allergens, we need to clean the house dust mite thoroughly. Asthma can cause recurrent episodes of wheezing, shortness of breath, chest tightness, and/or coughing. In addition, these emergencies often occur at night/morning. Therefore, this special situation requires us to pay close attention to the patient’s changes during the course of nursing.

In conclusion, our research utilized the PDGF-induced ASMCs to investigate the function and mechanism of YTHDF1 in asthma. These findings revealed the regulation of YTHDF1 on ASMCs’ proliferation and migration. Mechanistically, YTHDF1 significantly combined with cyclin D1 mRNA, thereby enhancing its mRNA stability through m^6^A-depedent manner (Fig. 6). Overall, this study may provide new insight for asthma.

##  Supplemental Information

10.7717/peerj.14951/supp-1 Supplemental Information 1 Uncropped blots

10.7717/peerj.14951/supp-2 Supplemental Information 2 Primers

10.7717/peerj.14951/supp-3 Supplemental Information 3 PCR data

## Additional Information and Declarations

### Data Availability

The following information was supplied regarding data availability: The raw data is available in the Supplemental Files.
