# Supplementary figures and images for: N6-methyladenosine reader YTHDF1 regulates the proliferation and migration of airway smooth muscle cells through m6A/cyclin D1 in asthma
Source: PeerJ. 2023 Mar 24;11:e14951. doi: 10.7717/peerj.14951 (PMC10042154; doi:10.7717/peerj.14951)

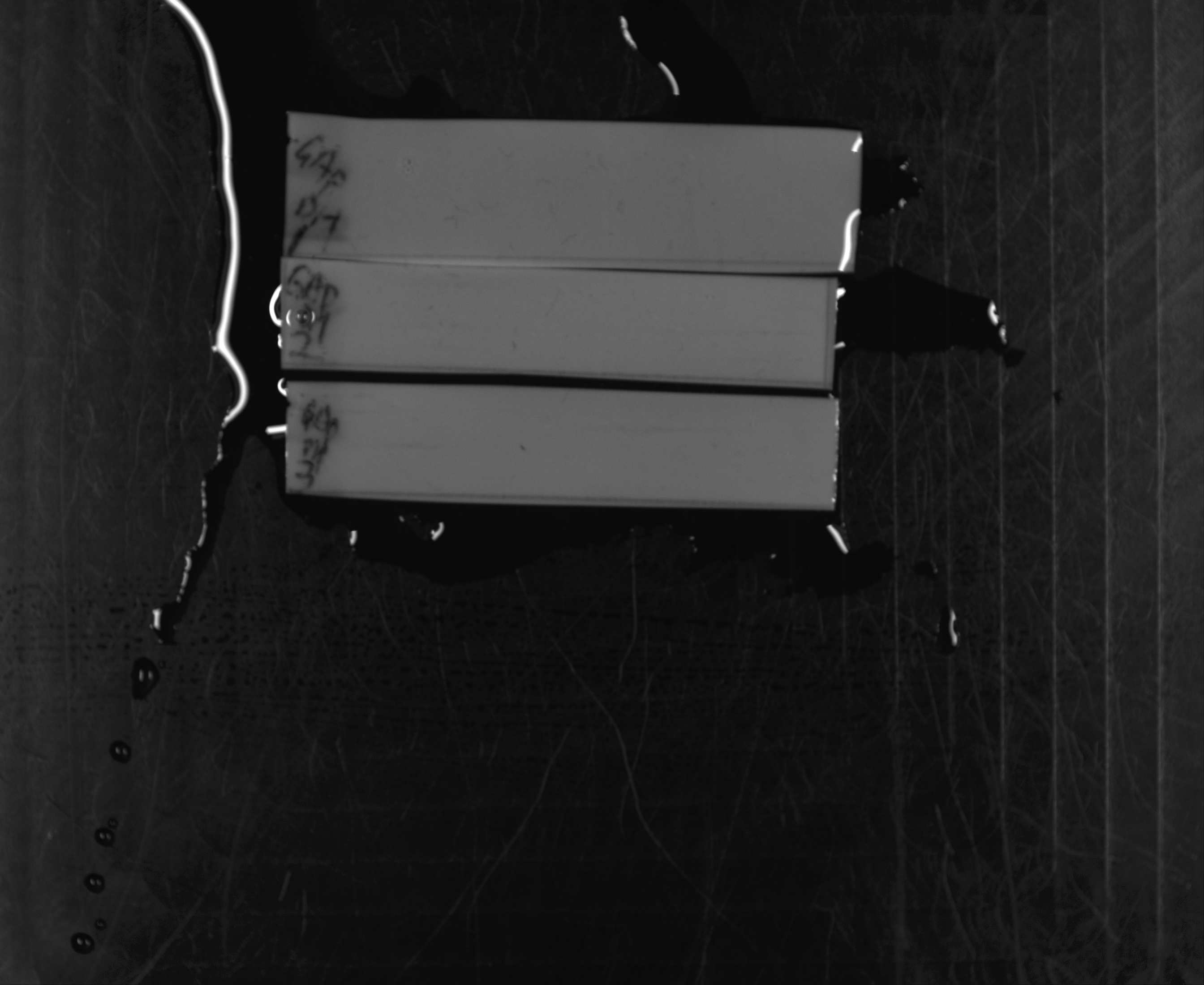

Supplement: Supplemental Information 1 [file peerj-11-14951-s001.zip › Uncropped blots/Fig 1B-2 .Tif]

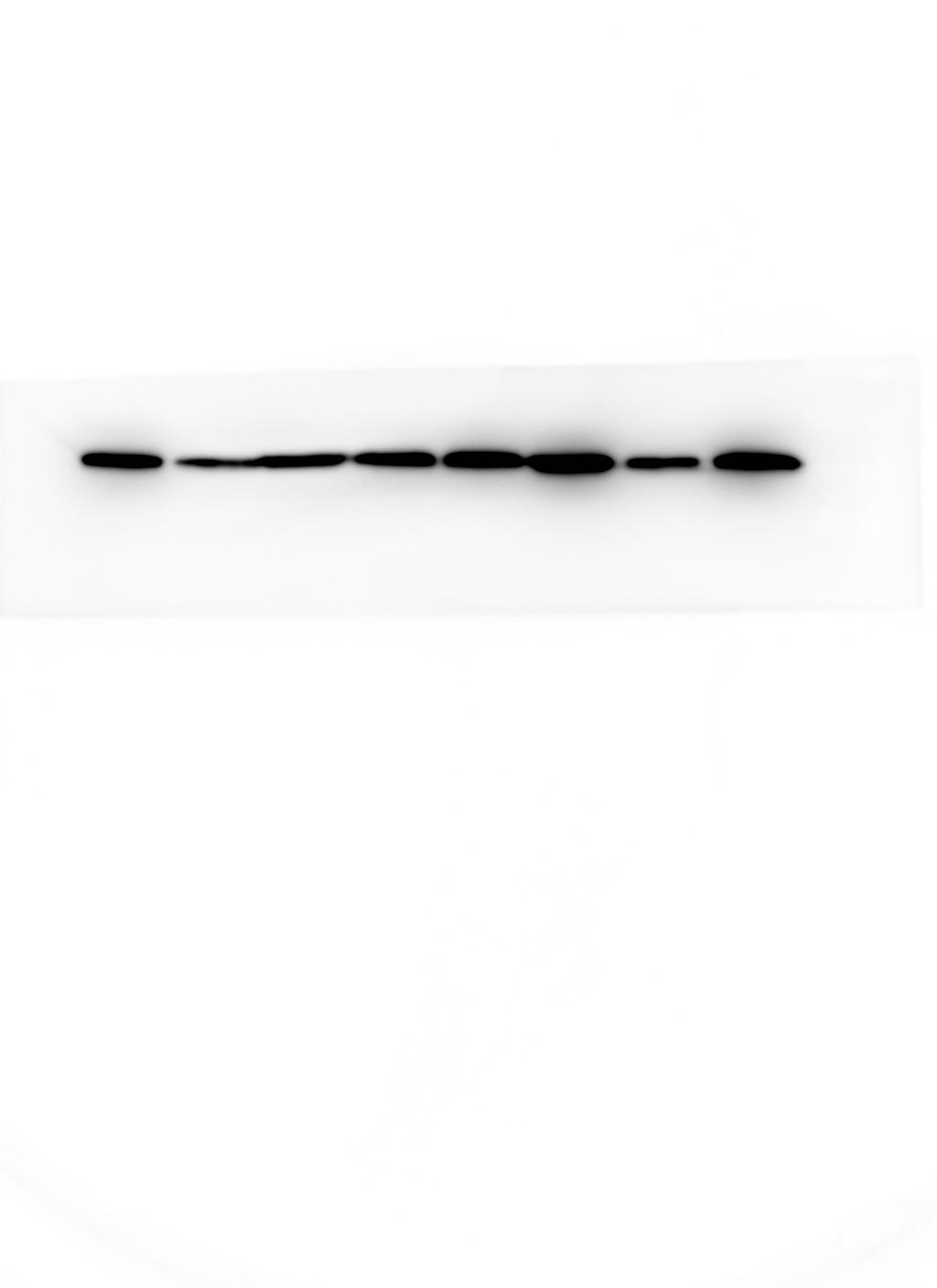

Supplement: Supplemental Information 1 [file peerj-11-14951-s001.zip › Uncropped blots/YTHDF1 blot -2 .jpg]

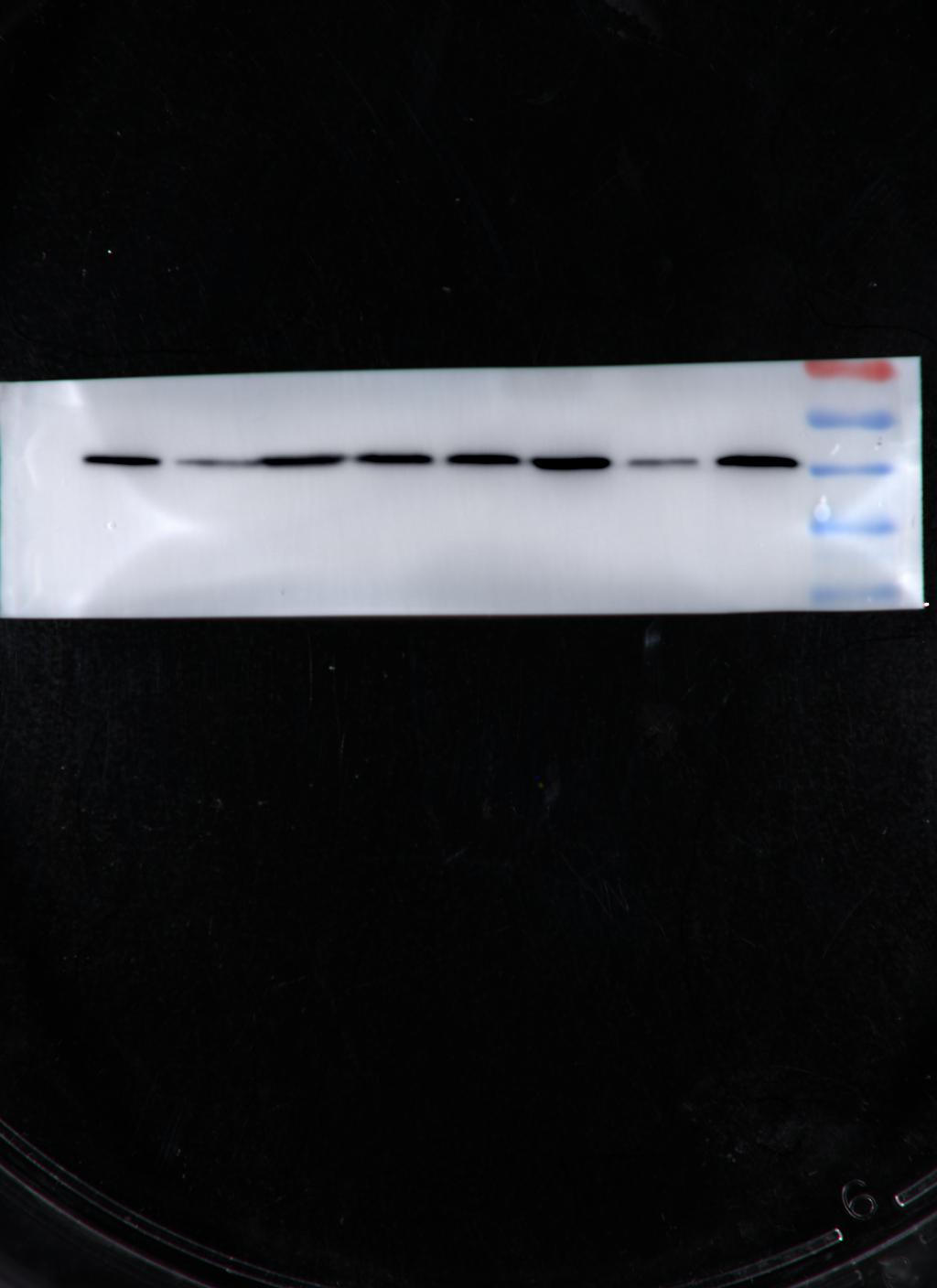

Supplement: Supplemental Information 1 [file peerj-11-14951-s001.zip › Uncropped blots/YTHDF1 blot .jpg]

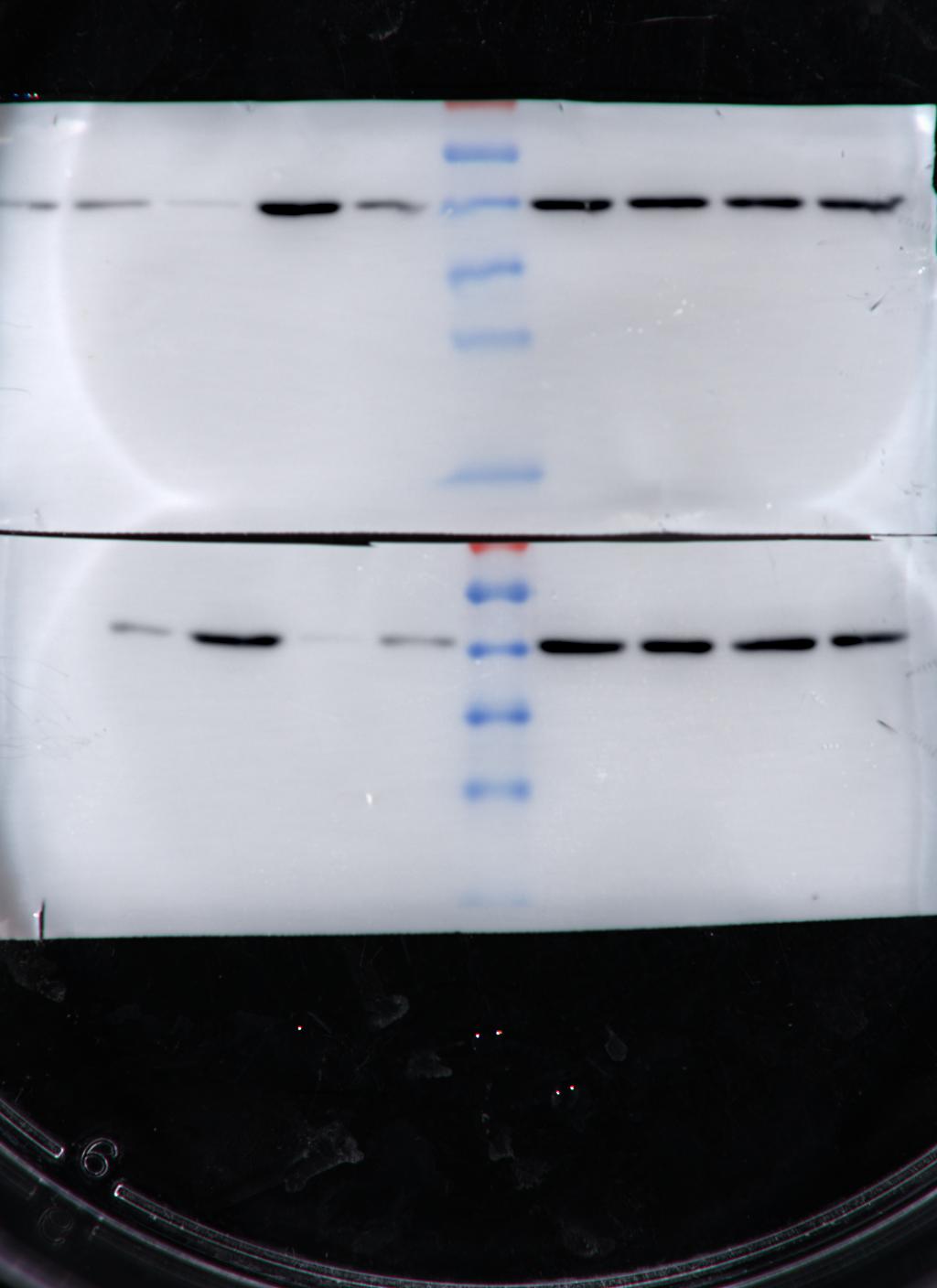

Supplement: Supplemental Information 1 [file peerj-11-14951-s001.zip › Uncropped blots/actin .jpg]
