# Supplementary material for: N6-methyladenosine reader YTHDF1 regulates the proliferation and migration of airway smooth muscle cells through m6A/cyclin D1 in asthma
Source: PeerJ. 2023 Mar 24;11:e14951. doi: 10.7717/peerj.14951 (PMC10042154; doi:10.7717/peerj.14951)
Supplement: Supplemental Information 2 [file peerj-11-14951-s002.docx]

**Table S1**. Primers sequences for qRT-PCR and sequences of shRNA.

|  | Sequences |
| --- | --- |
| YTHDF1 | Sense, 5’-ACCTGTCCAGCTATTACCCG-3’  Anti-sense, 5’-TGGTGAGGTATGGAATCGGAG-3’ |
| Cyclin D1 | Sense, 5’-GCTGCGAAGTGGAAACCATC-3’  Anti-sense, 5’-CCTCCTTCTGCACACATTTGAA-3’ |
| sh-YTHDF1-1 | 5’-CCCGAAAGAGTTTGAGTGGAA-3’ |
| sh-YTHDF1-2 | 5’-GTTCGTTACATCAGAAGGATA-3’ |
| actin | forward, 5’-CATGTACGTTGCTATCCAGGC-3’  reverse, 5’-CTCCTTAATGTCACGCACGAT-3’ |
