# Supplementary material for: N6-methyladenosine reader YTHDF1 regulates the proliferation and migration of airway smooth muscle cells through m6A/cyclin D1 in asthma
Source: PeerJ. 2023 Mar 24;11:e14951. doi: 10.7717/peerj.14951 (PMC10042154; doi:10.7717/peerj.14951)
Supplement: Supplemental Information 3 [file peerj-11-14951-s003.zip › PCR data/CCND1 UCSC .docx]

GACTGCCTCCGGGCCTGCCAGGAGCAGATCGAAGCCC

TGCTGGAGTCAAGCCTGCGCCAGGCCCAGCAGAACATGGACCCCAAGGCC

GCCGAGGAGGAGGAAGAGGAGGAGGAGGAGGTGGACCTGGCTTGCACACC

CACCGACGTGCGGGACGTGGACATCTGAGGGCGCCAGGCAGGCGGGCGCC

ACCGCCACCCGCAGCGAGGGCGGAGCCGGCCCCAGGTGCTCCCCTGACAG

TCCCTCCTCTCCGGAGCATTTTGATACCAGAAGGGAAAGCTTCATTCTCC

TTGTTGTTGGTTGTTTTTTCCTTTGCTCTTTCCCCCTTCCATCTCTGACT

TAAGCAAAAGAAAAAGATTACCCAAAAACTGTCTTTAAAAGAGAGAGAGA

GAAAAAAAAAATAGTATTTGCATAACCCTGAGCGGTGGGGGAGGAGGGTT

GTGCTACAGATGATAGAGGATTTTATACCCCAATAATCAACTCGTTTTTA

TATTAATGTACTTGTTTCTCTGTTGTAAGAATAGGCATTAACACAAAGGA

GGCGTCTCGGGAGAGGATTAGGTTCCATCCTTTACGTGTTTAAAAAAAAG

CATAAAAACATTTTAAAAACATAGAAAAATTCAGCAAACCATTTTTAAAG

TAGAAGAGGGTTTTAGGTAGAAAAACATATTCTTGTGCTTTTCCTGATAA

AGCACAGCTGTAGTGGGGTTCTAGGCATCTCTGTACTTTGCTTGCTCATA

TGCATGTAGTCACTTTATAAGTCATTGTATGTTATTATATTCCGTAGGTA

GATGTGTAACCTCTTCACCTTATTCATGGCTGAAGTCACCTCTTGGTTAC

AGTAGCGTAGCGTGCCCGTGTGCATGTCCTTTGCGCCTGTGACCACCACC

CCAACAAACCATCCAGTGACAAACCATCCAGTGGAGGTTTGTCGGGCACC

AGCCAGCGTAGCAGGGTCGGGAAAGGCCACCTGTCCCACTCCTACGATAC

GCTACTATAAAGAGAAGACGAAATAGTGACATAATATATTCTATTTTTAT

ACTCTTCCTATTTTTGTAGTGACCTGTTTATGAGATGCTGGTTTTCTACC

CAACGGCCCTGCAGCCAGCTCACGTCCAGGTTCAACCCACAGCTACTTGG

TTTGTGTTCTTCTTCATATTCTAAAACCATTCCATTTCCAAGCACTTTCA

GTCCAATAGGTGTAGGAAATAGCGCTGTTTTTGTTGTGTGTGCAGGGAGG

GCAGTTTTCTAATGGAATGGTTTGGGAATATCCATGTACTTGTTTGCAAG

CAGGACTTTGAGGCAAGTGTGGGCCACTGTGGTGGCAGTGGAGGTGGGGT

GTTTGGGAGGCTGCGTGCCAGTCAAGAAGAAAAAGGTTTGCATTCTCACA

TTGCCAGGATGATAAGTTCCTTTCCTTTTCTTTAAAGAAGTTGAAGTTTA

GGAATCCTTTGGTGCCAACTGGTGTTTGAAAGTAGGGACCTCAGAGGTTT

ACCTAGAGAACAGGTGGTTTTTAAGGGTTATCTTAGATGTTTCACACCGG

AAGGTTTTTAAACACTAAAATATATAATTTATAGTTAAGGCTAAAAAGTA

TATTTATTGCAGAGGATGTTCATAAGGCCAGTATGATTTATAAATGCAAT

CTCCCCTTGATTTAAACACACAGATACACACACACACACACACACACACA

AACCTTCTGCCTTTGATGTTACAGATTTAATACAGTTTATTTTTAAAGAT

AGATCCTTTTATAGGTGAGAAAAAAACAATCTGGAAGAAAAAAACCACAC

AAAGACATTGATTCAGCCTGTTTGGCGTTTCCCAGAGTCATCTGATTGGA

CAGGCATGGGTGCAAGGAAAATTAGGGTACTCAACCTAAGTTCGGTTCCG

ATGAATTCTTATCCCCTGCCCCTTCCTTTAAAAAACTTAGTGACAAAATA

GACAATTTGCACATCTTGGCTATGTAATTCTTGTAATTTTTATTTAGGAA

GTGTTGAAGGGAGGTGGCAAGAGTGTGGAGGCTGACGTGTGAGGGAGGAC

AGGCGGGAGGAGGTGTGAGGAGGAGGCTCCCGAGGGGAAGGGGCGGTGCC

CACACCGGGGACAGGCCGCAGCTCCATTTTCTTATTGCGCTGCTACCGTT

GACTTCCAGGCACGGTTTGGAAATATTCACATCGCTTCTGTGTATCTCTT

TCACATTGTTTGCTGCTATTGGAGGATCAGTTTTTTGTTTTACAATGTCA

TATACTGCCATGTACTAGTTTTAGTTTTCTCTTAGAACATTGTATTACAG

ATGCCTTTTTTGTAGTTTTTTTTTTTTTTATGTGATCAATTTTGACTTAA

TGTGATTACTGCTCTATTCCAAAAAGGTTGCTGTTTCACAATACCTCATG

CTTCACTTAGCCATGGTGGACCCAGCGGGCAGGTTCTGCCTGCTTTGGCG

GGCAGACACGCGGGCGCGATCCCACACAGGCTGGCGGGGGCCGGCCCCGA

GGCCGCGTGCGTGAGAACCGCGCCGGTGTCCCCAGAGACCAGGCTGTGTC

CCTCTTCTCTTCCCTGCGCCTGTGATGCTGGGCACTTCATCTGATCGGGG

GCGTAGCATCATAGTAGTTTTTACAGCTGTGTTATTCTTTGCGTGTAGCT

ATGGAAGTTGCATAATTATTATTATTATTATTATAACAAGTGTGTCTTAC

GTGCCACCACGGCGTTGTACCTGTAGGACTCTCATTCGGGATGATTGGAA

TAGCTTCTGGAATTTGTTCAAGTTTTGGGTATGTTTAATCTGTTATGTAC

TAGTGTTCTGTTTGTTATTGTTTTGTTAATTACACCATAATGCTAATTTA

AAGAGACTCCAAATCTCAATGAAGCCAGCTCACAGTGCTGTGTGCCCCGG

TCACCTAGCAAGCTGCCGAACCAAAAGAATTTGCACCCCGCTGCGGGCCC

ACGTGGTTGGGGCCCTGCCCTGGCAGGGTCATCCTGTGCTCGGAGGCCAT

CTCGGGCACAGGCCCACCCCGCCCCACCCCTCCAGAACACGGCTCACGCT

TACCTCAACCATCCTGGCTGCGGCGTCTGTCTGAACCACGCGGGGGCCTT

GAGGGACGCTTTGTCTGTCGTGATGGGGCAAGGGCACAAGTCCTGGATGT

TGTGTGTATCGAGAGGCCAAAGGCTGGTGGCAAGTGCACGGGGCACAGCG

GAGTCTGTCCTGTGACGCGCAAGTCTGAGGGTCTGGGCGGCGGGCGGCTG

GGTCTGTGCATTTCTGGTTGCACCGCGGCGCTTCCCAGCACCAACATGTA

ACCGGCATGTTTCCAGCAGAAGACAAAAAGACAAACATGAAAGTCTAGAA

ATAAAACTGGTAAAACCCCA

>hg19_knownGene_uc001opa.3 range=chr11:69455873-69469242 5'pad=0 3'pad=0 strand=+ repeatMasking=none

CACACGGACTACAGGGGAGTTTTGTTGAAGTTGCAAAGTCCTGGAGCCTC

CAGAGGGCTGTCGGCGCAGTAGCAGCGAGCAGCAGAGTCCGCACGCTCCG

GCGAGGGGCAGAAGAGCGCGAGGGAGCGCGGGGCAGCAGAAGCGAGAGCC

GAGCGCGGACCCAGCCAGGACCCACAGCCCTCCCCAGCTGCCCAGGAAGA

GCCCCAGCCATGGAACACCAGCTCCTGTGCTGCGAAGTGGAAACCATCCG

CCGCGCGTACCCCGATGCCAACCTCCTCAACGACCGGGTGCTGCGGGCCA

TGCTGAAGGCGGAGGAGACCTGCGCGCCCTCGGTGTCCTACTTCAAATGT

GTGCAGAAGGAGGTCCTGCCGTCCATGCGGAAGATCGTCGCCACCTGGAT

GCTGGAGgtgcggggcttcgggcggctctcttaagacttccctgcaactt

gttgcccagacccacgtttctttgctactcacccccctcccttctctccc

gctagaactttgaagtttgccgtggtgtttctagggatccgtattttcaa

aataaaaattgcgggtattttctgaaggaggaaggggtgggggtgggggt

gctagaagtagcgtttcgtgggaggggagaagggggtccgggaggggtgc

cttcgggagaagccagtgccaggggcaccccaatgggcccgagggtgcgg

gctggcaggctgggtgcgctttgtgtcccccgcctgcgccccagcccggc

tgcgcctcagcggccgggagccgccaactccggggggagggggcatagat

ttgatttttaaattaatatccatggacacgtatgcaagggccgctcgtgc

cagtattatgcgccatctttgctcttttattgcaaagcaaaagtgtttat

taataattgggggcagggtgggggcggggagcggccgccgggcgctgggg

ccgcagctaagggccgcgcggctgccgggagcccgcgggaggggcgcagg

gacgcggcatgggtagttttggggggacgccgctagggaagggggggcct

ttgttcaagcagcgagtcccggggcgccccgaacgggcagcctgggccgg

agagcacggcgagctgcaaggtcgcgtggcccccaagacgccagggcttg

atccccgtctgcagggatatcggcttggaggaccttctccgagcgagccg

ggggcctgggagcacattttcagaccttcggtgggcgcctgaggggcccg

caagtattttaaaataatttttgaaagtgcggcgtggtgcccttgcgaga

gggaaacgccgcccgcgcccagggggaagggggggccccggagtttgaat

tcctggggctccccccggagcctgtaacgaactcccaacccccggcctgg

gtaaagggtcgcccgagggtcattttcagggtttttttatgcacttagtt

atttttttaatatttttaaatattttttgaaaagatgacgtctggggaaa

tgcggcgcggcggcctgggacgccacctttgtgtctcgcaggcgcggcgc

ccaaccccgcggcccgttccgcggccccgcaccccagttggtgtcgaccc

ccagtcagagggaccacggagctccagggcgggccagggtcccgggggcc

ggcagcccgcgccgccgcgcacgccgcccagctgtgcccgctcccgcccc

caccgtgccagcctcgcggggactttccctttcagtttcggggagggtgg

gtactggggacgcgcgggggagggggcgcatcacgggaagctcctgccgc

ccccagccccgacccctcggcgccctccagacctggcggccctgccaagc

gcgatggggggtgcgggggcgtgcgggggggcggcgcgacctggcggcgg

cggtcacgggccccgtgcctccgtagGTCTGCGAGGAACAGAAGTGCGAG

GAGGAGGTCTTCCCGCTGGCCATGAACTACCTGGACCGCTTCCTGTCGCT

GGAGCCCGTGAAAAAGAGCCGCCTGCAGCTGCTGGGGGCCACTTGCATGT

TCGTGGCCTCTAAGATGAAGGAGACCATCCCCCTGACGGCCGAGAAGCTG

TGCATCTACACCGACAACTCCATCCGGCCCGAGGAGCTGCTGgtaaccac

tggaccccgccgccccccgccccccgcgagccgcacgcaggaccacgggg

ccggggaaggtgcaggcggtggcggccggcccgcctctgacatatctgct

cctccgagggagggcggccccgccgccgggcgtccctgtccggggagcgg

gcgggatcctagccgccctcgtcccgccgccctgtgtgcgcttgcctgcg

actcccaccgcgttcgcgccccgcggtgtggccgaaaagtgggcggcgcg

cgccctccagcggctgcacgaggagcgccgcgctcggcgctgagcctcca

gttccaggtggtgggaggtctttttgtttccacttgcagagtcttttcac

gcggcgggcgccttttctgttttgatctgggattgcgtgttgccccagct

cccttgagtccccagcattcgccagccctcccctccaacatccaggaccg

cacgagacgcaggggccagtgctctgagccggaggtgcggcgtggcccgg

cccccgtgctgccggcttccccgcgcccccgggctggcccgcacctcccc

tgatggccgctcaccctgtgttcgcagCAAATGGAGCTGCTCCTGGTGAA

CAAGCTCAAGTGGAACCTGGCCGCAATGACCCCGCACGATTTCATTGAAC

ACTTCCTCTCCAAAATGCCAGAGGCGGAGGAGAACAAACAGATCATCCGC

AAACACGCGCAGACCTTCGTTGCCCTCTGTGCCACAGgtagggcaggccc

ggcagcccccggcctccccttgagagccggctccttaggtgaccctggcc

ggcttcttgctctccacctgggtgctgtctgggaagatgtccccagaccc

cctcctgcgctggagagcgctcttccagctctggtgagcagaggccctgg

attgtttgtcgcgctggatggagggagatttgctccctcacggccaccat

gcagtaccttgggcattggtgtggacggctcagcctgcctgtgtcccgtt

actctggcctcgtccttcaggccaggcagcctgtggccactccatgctga

aaggggtttaccttggccacagggccgcctcctttctccacccacctcca

gcccttcttgtgtccttaaggagcctgagctgcagaggccccctcctggc

ctctcccaggctgggccacctgccagaggcgcctccaggggcggggagag

ctgtcggcctgcctgcaccacgtgctctgggcagccgagtgcaggggtgt

ccagcagaggagctcggctgcctgaggccctgccaggggtgccggcagcc

agccgggctcagctgagccctgagggggcgcttcagagcactctcagctt

gggccgccaccgtgggcagcagaagcacccagtcctcacttcccctggca

tggccccagaggcccctccctgacatggccttggccccagaacccagtgg

ggacagactcgcacatacacagggtgccgcctcctgctgtccccagccct

gcctctgacccccctgtgaccgcctccttccctggcccaggaggcctggt

taccttcatgggggagcatggccccatcccacccagctctgctgtggccc

acctttggtcaagcctcagttgtcacatctgtttgggggctcactctggg

tgacctaggccacaaggcccacggggcatcaaagaggcagtagcatcttc

tcccctccccagagggcagagccccccaagcctacttcagagctcccttc

tgacaccggtagcccgcagccggtattccagaatgggttctggtttaggc

gtgaggcctcccccacctcctccacctgcttggggcatgaacccctcccc

cacgtttccaagcgagtccccaaggtgggcagatgaagatgccaaggatg

tcgaccagtctggatgggtctggggtgggggggcatgcggcagacaggga

ggcattctctggctggtgctcctcagaggagagaggcctccggagactcc

agacagccttttatggagctgaaagtggcttcagagaaatgcaaagtttc

ctggagagaacgtggggcgtggttcttgcacagcctccctacagggtggc

tccagcagtggagctcccctcccaggacccctgggtgctagtgggaggca

gtgggcaggtgcagattctcgtccttcccactactgcacaccctttgtct

gcgaaggcgcccccagcggtgggtgaaggaggagggacacttggggaccc

agctgtgcacgtgctctcagtgactgtggagtccactccagggtgggtcc

cgagggaggggcaggagaccaggggacccacccctgcaaagtgctccggg

tcctgacccgtggccaccccatggaacgtaactgagcagccagtgccttg

ttcctgctggacatctgtggagacaagagtgacttacggctgcttaaagt

cagaaacaggttgaaggaggtggaggcgtgggaaagagtctaggaaggtg

tttttgccctccacgtggcaaaggttacatttaaaggtgatgctgggtgt

tctccctgcactaggcattcctggccccaggtccccagcaggtgtgcaca

tgctgcatacactcacgcatgggggtttcagggcaggtgcgcccttggct

ccgtgggaggccaggtgaggaacgtccagtgccaaggagcttccgggaca

gctgtcacttccctttacaaccaggcagcggatagggtcaaatcctggag

ctttggtgtctaattctgggtggctcctaatctaagcacagacagcacca

cacactggggtgggggcacgagcttctgaaacaacgtggccccagtgact

ccacgctgtgtgtgcccctggagacgggggggtgcacaaggtgcggagcc

agctagaacctgtcgctccctgcagaagcggtttctgtgtgcggttctga

tttgcctcaatgagaaggttttcattcatggctcccggctctcagactgg

gtggaactgctcccatttaaaggggaaaagaggtggctcggctcgttaag

gatttctttttctaagttgttacggcgcccagcagccggctttgtctccc

cttcagggtggctgcctttcttcccggcccctcgccggcggccctctctt

taacaaggccgaagttgtttattctctcgggatgaagtctcggatgggcc

gccacacccctggcggcccgtgggggcccctctccctttgtgcctgggtc

ggctcccattcagctcccccgaccccccttgttcccgggcgctcagtggc

gcgagatgaggcgatggggccgacaaagatgccacactcatccctgccga

cgtccggctcccagcccagggcccctggttcctgtgcagaattcctcgtg

ggtgtgacaaaaggctgcccccaggctccgctggggtgggggccaggcca

agaggcacatcccacactggcccacctgtccacggtaggcgcatgactgc

cctgaggaggggaggccggcattccccgccacaaaccaggacgtaattgg

tggcagggctctctgtggaaagagccagtctgctgtttgtctaggaggtc

agtcacagaggccccgagacgcccactactgcagcctggcaggcggatga

gcccagtatctggcagtgaccagagggagttttgtgcagaccacaaaggc

tgatgggccgccctagattggtgtccctcttggaagtgggcccagatgtg

cgggacagtccccaggaagccccaggtgagggcactggtgccctcttggg

aaagctgctccctcctggggcccggctcccggcccagtcctccaggggtg

tcccatggtgactggtgctaggaaccccacacctcttcccttacttggga

agtcactggaattgttgggctacatcagacggcccagaaaagtgtttttg

tcatcggccagaaataggagagttgtgagtagagggcccgggtggagttg

gggtgtacttggtctgtgctctgaaggtcactgtgacagtcatggtccca

tggtaaggggcatgggttgctggaagagctcttccttcccgagtgagcca

agccgggctctcctggcgccagggcctgagccgcagccacaccacagccg

ccctgaaggctgccggccagggcttacccctcaagggacacggaatggct

tcatcagtaccctgcagccccgtggcctggcccgggtggaggcctaggct

tcagccatgcgatgtcccttcagaatatgacttgtctgcaatccctgctg

ctggggggtggcaggtacttggggtgagggttagggtcatagaagcgaca

tctctacgtcctcatatttgcgtcatctaattttgtttttgtgaatacgt

gataacattcacaaggctcaagatgctaaaaggatgagaaggcagtgatg

tccccatcacctgtcctgtgtcttcccgtggctttctctttccttggtta

tgtttgagtcaacagtggggctgacgttccaggagggtccgtgggccagg

ctcttgctctccgagtgcccagggatggctggaggctgaggagggcctgg

atgtggagcctcagataccgagtgcttcccttcaggccgggccgcttgct

cagagccagcacacagggatgcccggatcacgggggccctgagagggtcc

cctgctcacagcctccttccctctctccttctgcctcagATGTGAAGTTC

ATTTCCAATCCGCCCTCCATGGTGGCAGCGGGGAGCGTGGTGGCCGCAGT

GCAAGGCCTGAACCTGAGGAGCCCCAACAACTTCCTGTCCTACTACCGCC

TCACACGCTTCCTCTCCAGAGTGATCAAGTGTGACCCGgtaagtgagggt

gatgtcccaggcagccttgccggggcttacagggggagacacctagtgcc

acggaaatgccgaggctggtgccaaggcccccaagggtgacaaggttggg

gctggggctgggcccctcggaccccaggccacagactgacagggcaccgg

cttcttccactgctcctagaacttactgactggctgggaggtcctcacag

ccttctcacgtcccctggggcttccaggagccgtagagtttctgggcgaa

gcgtccgggacggaggccccaggcggccccagccaatggtctgtgtggtg

atggtgtgtggggttaggcccaggcgagctttgtttgggccacaatgtgc

gtggccaataaatagatgcttgaaaagggctcctgtgaggtccgagacac

cggacaacgggcggatagagacagccttgttgtttacggcctctttgaga

ggctgctgctgttaaaccctgggatgactgtgtctttcttcttaaaaatg

ccattgttttattcccgagtcttttcttaaagaaagaattaaaatgacaa

tcaaaagggtttgtggcatttaccaaattagaccagagaggtggccgggt

cagccgccggccccgcggtgtgtgagggagtgaccgcctgaccccagctt

ggggctgggtgggcctgcaagacccgttttggctctggcctgggccgcct

cttggtggtctgccctcgagcctcccggggactccgcacgggtctcagca

gatgctatctagggtccacctgcctgtcccctgcctagtggtgcctctgt

cccggggacactgggagtagcggctgcccagcccatgtgtgtctcggaag

aggaagaagcttttttgccgtgggacaccgaagttggcaggggcctccct

tctgtgttctcggccatggcctcccttgcaccctgccccgtgttatcctt

tgggggtggtgaggtgtcctcacccgctgtagggtggaggccagcagccc

gcagctctctcaggaaaatggctcagaaacaccatcgaggcctccagaag

cccagcaaagagaaagcccctccatcaaaatgaaactcgcgtctgcactt

ttcatttcgaactccacgccctgagtgaaaaccgcttccccgccaggggt

gactgccctgggatgttgctgtcttcgggcagttgtgggaagttgggcgc

tggcccttatttgagtagagaccatcttaactagattggaggcacacgtc

tcacagctgacagacacacggggtgaagttacccgaggcggagtccactc

tgcctgatcagctagtgaccaacgtagctgagcccagactcagaaaaacc

gtccacagcagaggcccctgcattttctagggcgtgttctagaattttct

ttggtgggtggaatgtccatctgtgcaaatcgggtgcgcagtgccacaca

ccagtgacttttcgcggaggagcgtgctgcctttttggagcttctggctg

tgggagaacagctttgtccaccggggtagccttgcaggcagctgtggggc

cagaggaatgaaggaaggtcctggagtctagctgcatgtgtgaccctgga

gtgggtcatgggcgagggacgggccgcaggtgaagaatccctggatggag

ctgccaggcccctggggctgagaattgaagctggctggtgttttaggttg

aacgtcaggagtcttgtatctcaccccaggcctctggcctcagtttcccc

atctgtacagtgggactgtttgtgcagccagcccggccagcttcatttgc

catgatgagaatttatctgaggggcgggagaggaaagccctccctataaa

ggtacaggcgctaaaatgtcgtgacctcagtggtccacctaaaagtcgtt

ctggcctgggtcatcgcctgtcgtgctatgcctttgtccagccccttctg

gttgggagttaagtggcacctgtgcggcacgtggtggggctgtggcccag

ccctgctccttgtggaaggtctgtttcctgggctgcctagagacttggct

tgaagccctagcgtggcttcctggcagttgggacacacacagccccaaca

catggagccggttctccatccagaagcccccgggcagtaagcagccactt

caggctgcgtgggacttgcccgtggtggagcctaggagaggcccctggct

gggcgtggcgttccagatttcacggctgctctttcccactgacagtgtgg

tgtggacgctgccaagggagtctggagccccagagggtggaggtgcagga

cttccaggagcgtccgtcgcactccacccgagggcgagcacctcagtggc

cgcagtgggtggatgcatgctgtgccaggctgatggctggccccggggca

caggcctgagcgggagaggatggaggggagggatcaatggtccaggtccc

cctggccacccagcattcatcctcagtcatgcacggcccaaggcttcgac

agccattgatcatggaaggccaggttcacctcaagggctgccacatggag

aggttaagtctgaaaaggctgaaaaggcagggttcaaagggcctcctgtc

cagatcagatggcactgaattccccagggagctggcacggccagtgggaa

caggcggtgaaggcgctgttggacatggggacgggcagggggtgtgcagg

gtgggcgggcaagcatctggtgtcttgtggctccagagaccaggtgggag

gtggaggcatttggtcctgagtgtcctgacaggtgatggcagctcccaca

tctcgctcaggttcagaggaggcagcatgggccgagggacagtttttggc

ttagtcttgctcttataaaggcttccgggtcatggcacctgggaaggggc

cctcgctgcaggccccttctaaggaccccctcttcccacctctccccacc

ctctctctctcagGACTGCCTCCGGGCCTGCCAGGAGCAGATCGAAGCCC

TGCTGGAGTCAAGCCTGCGCCAGGCCCAGCAGAACATGGACCCCAAGGCC

GCCGAGGAGGAGGAAGAGGAGGAGGAGGAGGTGGACCTGGCTTGCACACC

CACCGACGTGCGGGACGTGGACATCTGAGGGCGCCAGGCAGGCGGGCGCC

ACCGCCACCCGCAGCGAGGGCGGAGCCGGCCCCAGGTGCTCCCCTGACAG

TCCCTCCTCTCCGGAGCATTTTGATACCAGAAGGGAAAGCTTCATTCTCC

TTGTTGTTGGTTGTTTTTTCCTTTGCTCTTTCCCCCTTCCATCTCTGACT

TAAGCAAAAGAAAAAGATTACCCAAAAACTGTCTTTAAAAGAGAGAGAGA

GAAAAAAAAAATAGTATTTGCATAACCCTGAGCGGTGGGGGAGGAGGGTT

GTGCTACAGATGATAGAGGATTTTATACCCCAATAATCAACTCGTTTTTA

TATTAATGTACTTGTTTCTCTGTTGTAAGAATAGGCATTAACACAAAGGA

GGCGTCTCGGGAGAGGATTAGGTTCCATCCTTTACGTGTTTAAAAAAAAG

CATAAAAACATTTTAAAAACATAGAAAAATTCAGCAAACCATTTTTAAAG

TAGAAGAGGGTTTTAGGTAGAAAAACATATTCTTGTGCTTTTCCTGATAA

AGCACAGCTGTAGTGGGGTTCTAGGCATCTCTGTACTTTGCTTGCTCATA

TGCATGTAGTCACTTTATAAGTCATTGTATGTTATTATATTCCGTAGGTA

GATGTGTAACCTCTTCACCTTATTCATGGCTGAAGTCACCTCTTGGTTAC

AGTAGCGTAGCGTGCCCGTGTGCATGTCCTTTGCGCCTGTGACCACCACC

CCAACAAACCATCCAGTGACAAACCATCCAGTGGAGGTTTGTCGGGCACC

AGCCAGCGTAGCAGGGTCGGGAAAGGCCACCTGTCCCACTCCTACGATAC

GCTACTATAAAGAGAAGACGAAATAGTGACATAATATATTCTATTTTTAT

ACTCTTCCTATTTTTGTAGTGACCTGTTTATGAGATGCTGGTTTTCTACC

CAACGGCCCTGCAGCCAGCTCACGTCCAGGTTCAACCCACAGCTACTTGG

TTTGTGTTCTTCTTCATATTCTAAAACCATTCCATTTCCAAGCACTTTCA

GTCCAATAGGTGTAGGAAATAGCGCTGTTTTTGTTGTGTGTGCAGGGAGG

GCAGTTTTCTAATGGAATGGTTTGGGAATATCCATGTACTTGTTTGCAAG

CAGGACTTTGAGGCAAGTGTGGGCCACTGTGGTGGCAGTGGAGGTGGGGT

GTTTGGGAGGCTGCGTGCCAGTCAAGAAGAAAAAGGTTTGCATTCTCACA

TTGCCAGGATGATAAGTTCCTTTCCTTTTCTTTAAAGAAGTTGAAGTTTA

GGAATCCTTTGGTGCCAACTGGTGTTTGAAAGTAGGGACCTCAGAGGTTT

ACCTAGAGAACAGGTGGTTTTTAAGGGTTATCTTAGATGTTTCACACCGG

AAGGTTTTTAAACACTAAAATATATAATTTATAGTTAAGGCTAAAAAGTA

TATTTATTGCAGAGGATGTTCATAAGGCCAGTATGATTTATAAATGCAAT

CTCCCCTTGATTTAAACACACAGATACACACACACACACACACACACACA

AACCTTCTGCCTTTGATGTTACAGATTTAATACAGTTTATTTTTAAAGAT

AGATCCTTTTATAGGTGAGAAAAAAACAATCTGGAAGAAAAAAACCACAC

AAAGACATTGATTCAGCCTGTTTGGCGTTTCCCAGAGTCATCTGATTGGA

CAGGCATGGGTGCAAGGAAAATTAGGGTACTCAACCTAAGTTCGGTTCCG

ATGAATTCTTATCCCCTGCCCCTTCCTTTAAAAAACTTAGTGACAAAATA

GACAATTTGCACATCTTGGCTATGTAATTCTTGTAATTTTTATTTAGGAA

GTGTTGAAGGGAGGTGGCAAGAGTGTGGAGGCTGACGTGTGAGGGAGGAC

AGGCGGGAGGAGGTGTGAGGAGGAGGCTCCCGAGGGGAAGGGGCGGTGCC

CACACCGGGGACAGGCCGCAGCTCCATTTTCTTATTGCGCTGCTACCGTT

GACTTCCAGGCACGGTTTGGAAATATTCACATCGCTTCTGTGTATCTCTT

TCACATTGTTTGCTGCTATTGGAGGATCAGTTTTTTGTTTTACAATGTCA

TATACTGCCATGTACTAGTTTTAGTTTTCTCTTAGAACATTGTATTACAG

ATGCCTTTTTTGTAGTTTTTTTTTTTTTTATGTGATCAATTTTGACTTAA

TGTGATTACTGCTCTATTCCAAAAAGGTTGCTGTTTCACAATACCTCATG

CTTCACTTAGCCATGGTGGACCCAGCGGGCAGGTTCTGCCTGCTTTGGCG

GGCAGACACGCGGGCGCGATCCCACACAGGCTGGCGGGGGCCGGCCCCGA

GGCCGCGTGCGTGAGAACCGCGCCGGTGTCCCCAGAGACCAGGCTGTGTC

CCTCTTCTCTTCCCTGCGCCTGTGATGCTGGGCACTTCATCTGATCGGGG

GCGTAGCATCATAGTAGTTTTTACAGCTGTGTTATTCTTTGCGTGTAGCT

ATGGAAGTTGCATAATTATTATTATTATTATTATAACAAGTGTGTCTTAC

GTGCCACCACGGCGTTGTACCTGTAGGACTCTCATTCGGGATGATTGGAA

TAGCTTCTGGAATTTGTTCAAGTTTTGGGTATGTTTAATCTGTTATGTAC

TAGTGTTCTGTTTGTTATTGTTTTGTTAATTACACCATAATGCTAATTTA

AAGAGACTCCAAATCTCAATGAAGCCAGCTCACAGTGCTGTGTGCCCCGG

TCACCTAGCAAGCTGCCGAACCAAAAGAATTTGCACCCCGCTGCGGGCCC

ACGTGGTTGGGGCCCTGCCCTGGCAGGGTCATCCTGTGCTCGGAGGCCAT

CTCGGGCACAGGCCCACCCCGCCCCACCCCTCCAGAACACGGCTCACGCT

TACCTCAACCATCCTGGCTGCGGCGTCTGTCTGAACCACGCGGGGGCCTT

GAGGGACGCTTTGTCTGTCGTGATGGGGCAAGGGCACAAGTCCTGGATGT

TGTGTGTATCGAGAGGCCAAAGGCTGGTGGCAAGTGCACGGGGCACAGCG

GAGTCTGTCCTGTGACGCGCAAGTCTGAGGGTCTGGGCGGCGGGCGGCTG

GGTCTGTGCATTTCTGGTTGCACCGCGGCGCTTCCCAGCACCAACATGTA

ACCGGCATGTTTCCAGCAGAAGACAAAAAGACAAACATGAAAGTCTAGAA

ATAAAACTGGTAAAACCCCA
